# Should We Use Hyperbaric Oxygen for Carbon Monoxide Poisoning Management? A Network Meta-Analysis of Randomized Controlled Trials

**DOI:** 10.3390/healthcare10071311

**Published:** 2022-07-14

**Authors:** Yu-Wan Ho, Ping-Yen Chung, Sen-Kuang Hou, Ming-Long Chang, Yi-No Kang

**Affiliations:** 1 Department of Emergency Medicine, Taipei Medical University Hospital, Taipei 110, Taiwan; yu_wanmr@yahoo.com.tw (Y.-W.H.); 143040@h.tmu.edu.tw (P.-Y.C.); 992001@h.tmu.edu.tw (S.-K.H.); 2 Department of Emergency Medicine, School of Medicine, College of Medicine, Taipei Medical University, Taipei 110, Taiwan; 3 Evidence-Based Medicine Center, Wan Fang Hospital, Taipei Medical University, Taipei 116, Taiwan; 4 Research Center of Big Data and Meta-Analysis, Wan Fang Hospital, Taipei Medical University, Taipei 116, Taiwan; 5 Cochrane Taiwan, Taipei Medical University, Taipei 110, Taiwan

**Keywords:** carbon monoxide poisoning, hyperbaric oxygen, nervous system symptoms

## Abstract

Carbon monoxide (CO) poisoning is a public health issue in numerous countries. Oxygen supplementation is the standard and initial management for acute CO poisoning. Normobaric oxygen (NBO) and hyperbaric oxygen (HBO) therapies for CO poisoning have been discussed for several decades. NBO, one-session HBO, two-session HBO, and three-session HBO have not been clearly compared, although there are some syntheses. Therefore, this study aimed to provide an overview of various HBO therapies for CO poisoning. We searched online databases for randomized controlled trials (RCTs) on this topic, and two authors individually extracted data on characteristics, mortality, headache recovery, general fatigue, memory impairment, and difficulty concentrating. Outcomes were pooled using network meta-analysis. We included eight RCTs (*n* = 1785) that met our eligibility criteria. Pooled estimates showed that HBO had no better outcomes than NBO. Moreover, two-session HBO seemed to have a higher general fatigue rate than NBO, and compared with one-session HBO therapy, it had a higher fatigue rate (risk ratio (RR): 1.29, 95% confidence interval (CI): 1.03–1.62), memory impairment rate (RR = 1.80, 95% CI: 1.01–3.19), and concentration impairment rate (RR = 1.85, 95% CI: 1.19–2.89). HBO may be ineffective for patients with CO poisoning. Therefore, clinicians should consider the available treatment options carefully before recommending HBO to patients.

## 1. Introduction

Carbon monoxide (CO) poisoning is a public health and economic issue in several countries. In the United States, at least 430 deaths from accidental CO poisoning have been reported annually, and approximately 50,000 people visit the emergency department because of CO poisoning each year [1]. The most common symptoms are severe neurological problems including headache, dizziness, weakness, confusion, and even loss of conscious with memory loss [1]. These symptoms are accompanied by upset stomach, nausea with vomiting, chest pain, and abdominal pain. Moreover, symptoms of delayed neuropsychological sequelae may occur, including general fatigue, difficulty concentrating, lethargy, emotional lability, amnestic syndromes, insomnia, dementia, psychosis, chorea, apraxia, agnosia, peripheral neuropathy, and urinary incontinence [2].

Oxygen supplementation and normobaric oxygen (NBO) therapy administered using a nonrebreather mask are the standard and initial treatments for CO poisoning [2]. Hyperbaric oxygen (HBO) therapy is sometimes recommended for patients who have lost consciousness or have severe poisoning [2], and has even been applied to manage CO poisoning among pregnant women, children, as well as infants [3,4,5]. Although some guidelines and recommendations with different treatment protocols by various recommended atmosphere absolute, sessions, and duration have mentioned the roles of HBO therapy in managing CO poisoning in the past 10 years [6,7,8,9], more evidence is still needed to form the guidance of the best practice on this topic [10]. HBO appears to have the advantages of increased oxygen dissolution in the blood and accelerated CO elimination and the disadvantages of risks associated with transportation of the patient to a treatment center, hyperoxic seizures, and barotrauma [2]. A randomized controlled trial (RCT) by Scheinkestel concluded that HBO does not benefit and may worsen the outcome [11]. Two trials by Annane et al. also provided no evidence of the superiority of HBO over NBO [12,13]. Even the study by Hampson et al. provided the same conclusion of no significant difference in outcomes between HBO and NBO [14]. However, a RCT by Weaver et al. published in the *New England Journal of Medicine* found that HBO showed better outcomes in delaying neuropsychological sequelae [15].

In the latest meta-analysis, Wang et al. showed that HBO therapy significantly reduces the risk of memory impairment compared with NBO [16]. However, they did not include an earlier RCT by Annane et al. [13] and misclassified three-session HBO into two-session HBO in a trial on the *New England Journal of Medicine* [15,16]. Relevant evidence still cannot give a clear picture about the efficacy of HBO therapies for CO poisoning. Therefore, we performed this systematic review with the aim to provide an overview of HBO therapies for patients with CO poisoning. We updated this topic by conducting a network meta-analysis of available RCTs to evaluate the effect of NBO and HBO therapies on mortality, headache recovery, general fatigue, and neurologic sequelae in patients with CO poisoning.

## 2. Methods

This systematic review was performed in adherence to the Preferred Reporting Items for Systematic Reviews and Meta-Analyses guidelines in terms of evidence selection, quality assessment, data pooling, and study report [17]. Protocols of this synthesis were published on PROSPERO (CRD42020150728).

### 2.1. Eligibility Criteria and Evidence Selection

The authors first finalized the eligibility criteria for evidence selection prior to starting the comprehensive search. The primary inclusion criteria were as follows: (a) studies recruited patients with CO poisoning, (b) studies in which patients were treated using HBO, and (c) studies that were RCTs. CO poisoning and HBO are the two core elements in this synthesis; thus, we searched for studies using the relevant search terms of CO poisoning and HBO in New PubMed. Besides the study-type filter for RCTs, no filters were applied. Subsequently, the search strategy was adopted to identify relevant articles in Embase, the Cochrane Database of Systematic Review, and the Cochrane Central Register of Controlled Trials (Cochrane CENTRAL). Besides, gray literature and clinicaltrials.gov were manually checked for relevant ongoing RCTs. Reference lists of systematic reviews, meta-analyses, and RCTs were also reviewed for potentially eligible RCTs.

Two authors (YWH and PYC) conducted the final search for potential articles before November 2021 (Supplementary Materials S1). They also independently excluded articles after screening titles and abstracts. Subsequently, upon retrieving the full texts of the remaining articles, they also completed further review individually. They used the following exclusion criteria: (a) non-RCT and (b) gray literature without details of the trial design, baseline characteristics, or relevant outcomes. A third experienced author was responsible for resolving disagreements between the first two authors through discussion.

### 2.2. Data Extraction and Quality Assessment

After evidence selection, the two authors (YWH and PYC) individually extracted data on study design, trial characteristics, and outcomes. Using the Cochrane risk-of-bias tool, the study design was assessed, including randomization generation, allocation concealment, blinding of patients, blinding of study personnel, blinding of outcome assessors, and follow-up. They performed a quality assessment based on the relevant information of study design. The trial characteristics included location, inclusion years, treatments, sex, mean age, CO exposure time, baseline CO level, and number of patients in a coma at baseline. We analyzed the following outcomes: mortality rate, headache recovery rate, general fatigue rate, memory impairment rate, and difficulty concentrating rate. All of these outcomes were dichotomous, and the two authors extracted the number of events and total cases for mortality, general fatigue, memory impairment, and difficulty concentrating in each treatment arm. The difference between headache cases at baseline and after treatment was calculated to provide the headache recovery rate. An experienced author participated in data extraction and quality assessment if the two authors had any disagreement on the processes.

### 2.3. Data Synthesis and Analysis

To overcome multiple treatments for a specific condition in data synthesis, network meta-analysis has been developed by combining evidence from direct and indirect estimates [18]. Since HBO could be treated in various session strategies and some of them had no direct comparison in previous trials, a network meta-analysis is appropriate for this situation. Network meta-analysis can be carried out by frequentist and Bayesian models, while the two methods perform similar estimates [19,20]. The present synthesis used contrast-based network meta-analysis because the frequentist method is easily understood and commonly applied [21]. Similarity and transitivity are core assumptions for network meta-analysis [22]; the present study satisfied these assumptions by giving a specific PICO framework and keeping similarity among studies by including better study design (RCT) [18,22]. We further applied a statistical technique to assess whether our model violated the assumption of transitivity [23,24].

We used the RR for quantitative data synthesis through contrast-based network meta-analysis, as the outcomes were dichotomous. RRs were reported with 95% CIs. We further constructed the SUCRA to clarify the probability of the best effect of medical treatments using NBO or HBO. SUCRA provided a value between 0% and 100%, with a mean rank based on the rank probability of each comparator among the most effective treatment.

We also tested for inconsistency in each outcome, because consistency is an important assumption in network meta-analysis. We used the loop inconsistency test according to the Lu-Ades’ method and the design-by-treatment interaction model when an outcome was contributed by only a two-arm trial and trials with various arm designs, respectively. Moreover, we examined publication bias in the pooled estimates. Publication bias was detected using funnel plots with Egger’s regression intercept. Outcomes were interpreted carefully if any inconsistency or publication bias was detected. The abovementioned analyses were carried out using STATA version 14 for Microsoft Windows (StataCorp LP., College Station, TX, USA).

## 3. Results

**Figure 1:**
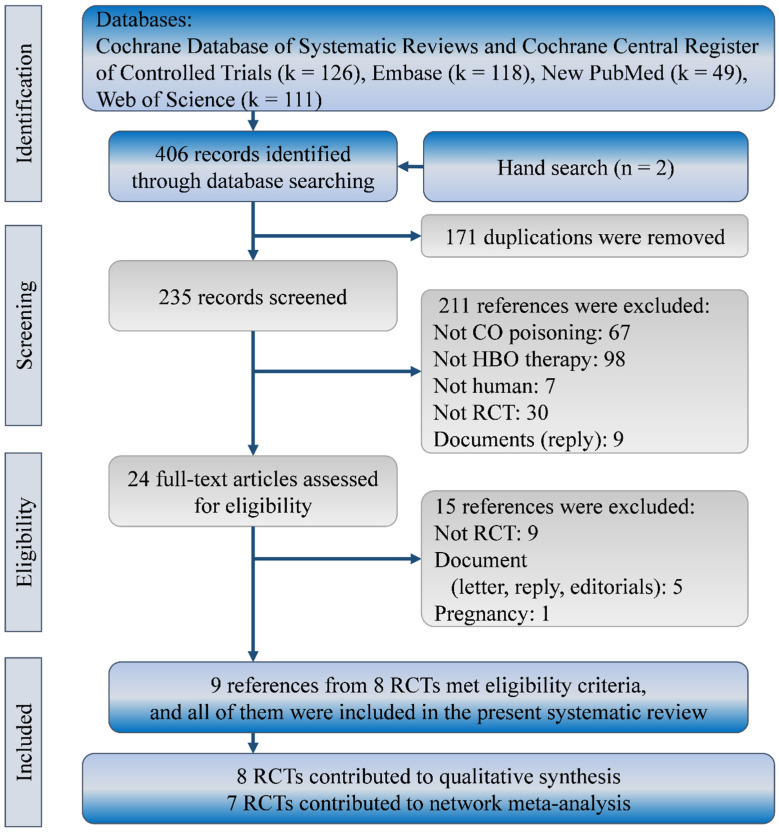
Flow diagram of study selection. RCT, randomized clinical trial.

Our search yielded a total of 404 references in the Cochrane Database of Systematic review (including CENTRAL; k = 126), Embase (k = 118), New PubMed (k = 49), and Web of Science (k = 111). No further published RCTs could be found according to trial numbers in clinicaltrial.gov. Two more references were identified from reference lists of relevant systematic reviews. We excluded 382 of them because of duplicated references (k = 171), irrelevant references (k = 172), non-RCTs (k = 30), and documents (k = 9). Of the 24 references that remained for further review, we further excluded 15 for non-RCTs (k = 9), documents (k = 5), and a special population (k = 1). Finally, we included nine full-text publications from eight RCTs in this systematic review with network meta-analysis [2,11,12,13,14,15,25,26,27]. Figure 1 shows the flow diagram for evidence selection of HBO treatments in patients with CO poisoning.

### 3.1. Characteristics and Quality of Included Studies

**Figure 2:**
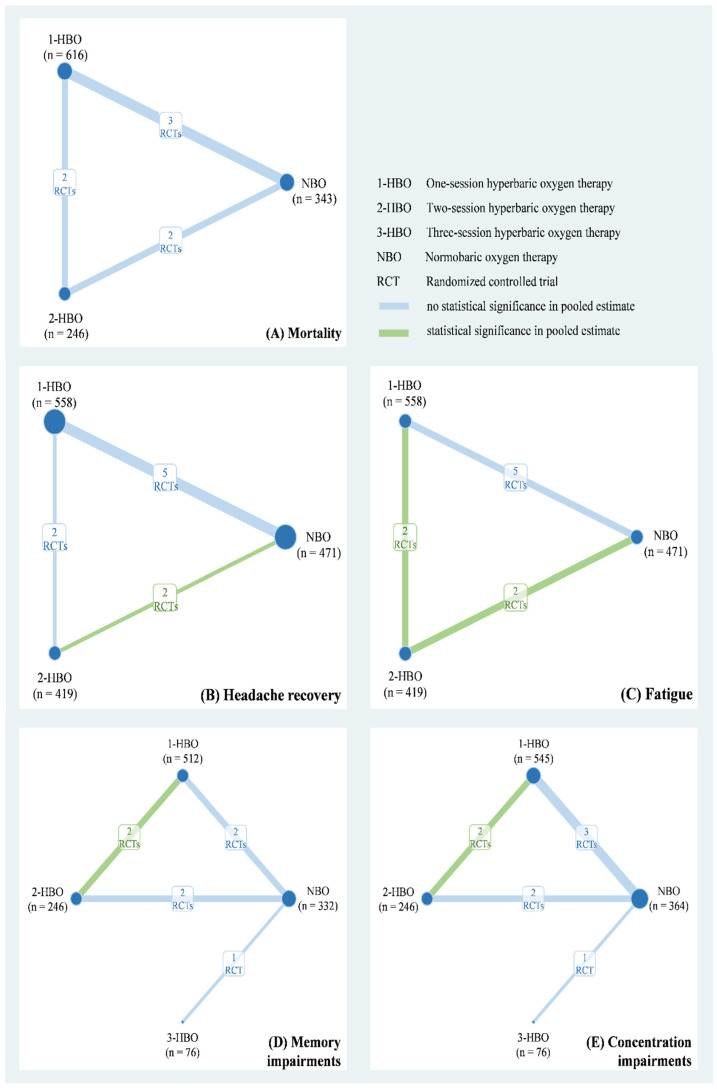
Network plot of hyperbaric oxygen strategies of each outcome.

The eight studies of our meta-analysis included a total of 1785 patients with CO poisoning from Europe and North America between 1989 and 2002. Their mean age ranged from 31 years to 49.7 years and included 929 men (52.04%). Table 1 shows relevant information on the duration of CO exposure, CO level, and the numbers of patients in a coma at baseline. The quality of the included RCTs is presented in Table S1. Six of eight studies were low risk for other bias and incomplete outcome data. Five of eight RCTs were low risk for sequence generation, allocation concealment, and selective reporting. However, seven of eight had either a high risk or some concerns regarding participant and study personnel blinding. Based on the available data, we applied a three-node network model with NBO, one-session HBO, two-session HBO treatments for the mortality rate (Figure 2A), headache recovery rate (Figure 2B), and general fatigue rate (Figure 2C). A four-node consistency model with NBO, one-session HBO, two-session HBO, and three-session HBO treatments was applied for the memory impairment (Figure 2D) and difficulty concentrating (Figure 2E) rates.

### 3.2. Mortality

**Figure 3:**
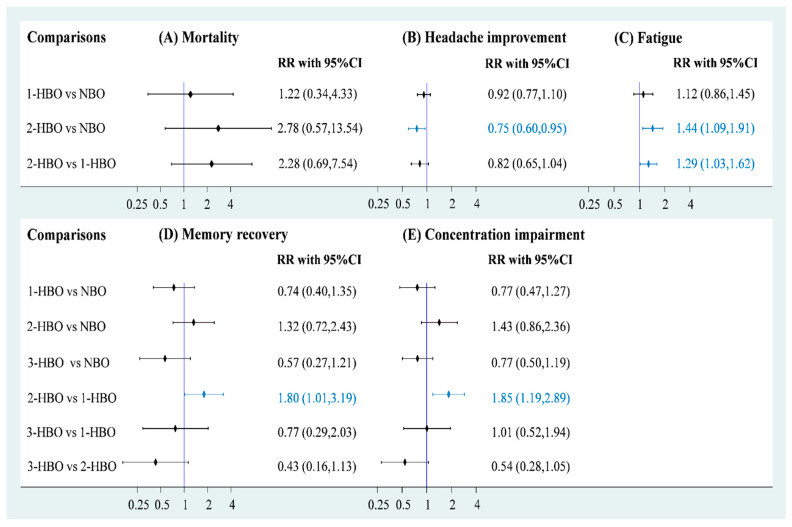
Forest plot of hyperbaric oxygen strategies of each outcome.

Data on mortality were available for three RCTs (*n* = 1205) to obtain a three-node network comprising NBO (*n* = 343), one-session HBO (*n* = 616), and two-session HBO (*n* = 246) treatments. The pooled estimate of mortality demonstrated that both one-session HBO (risk ratio [RR] = 1.34) and two-session HBO (RR = 2.78) treatments did not lead to significantly better outcomes in mortality rates than NBO treatment (Figure 3A). In addition, no significant difference was observed in the mortality rate between two- and one-session HBO treatments (RR = 2.28). We examined inconsistency using the design-by-treatment interaction model because the network meta-analysis of mortality included two- and three-arm trials; the inconsistency test showed no significance in the network model (*p =* 0.429; Figure S1). Furthermore, no significance was observed in the pooled estimate using Egger’s test (*p =* 0.086; Figure S2).

### 3.3. Headache Recovery and General Fatigue

Five RCTs (*n* = 1448) reported relevant information on headache recovery for NBO (*n* = 471), one-session HBO (*n* = 558), and two-session HBO (*n* = 419) treatments. Compared with NBO treatment, we observed no significant results for one-session HBO treatment for headache recovery (RR = 0.92). Two-session HBO treatment led to a significantly lower rate of headache recovery than NBO treatment (RR = 0.75, 95% CI: 0.60–0.95; Figure 3B). Moreover, no significant difference was observed in the rate of headache recovery between two-session HBO and one-session HBO treatments (RR = 0.82), and the two-session HBO seemed to be the worst management among the three interventions according to cumulative probability ranking (Figure S3). The inconsistency test conducted using the design-by-treatment interaction model showed no significance in the network model (*p =* 0.505; Figure S4). No significance was observed in the pooled estimate of headache recovery using Egger’s test (*p =* 0.772; Figure S5).

Two of the included RCTs (*n* = 1014) used a three-arm design and reported relevant information on fatigue. The data of fatigue were also included in a three-node network meta-analysis with NBO (*n* = 256), one-session HBO (*n* = 512), and two-session HBO (*n* = 246) treatments. We observed no significant difference in the rate of fatigue between one-session HBO and NBO treatments (RR = 1.12). However, a significantly higher rate of fatigue was noted for two-session HBO treatment than for NBO treatment (RR = 1.44, 95% confidence interval (CI): 1.09–1.91) and one-session HBO treatment (RR = 1.29, 95% CI: 1.03–1.62; Figure 3C). The surface under the cumulative ranking curve (SUCRA) analysis demonstrated that two-session HBO may be the worst treatment among the three interventions (Figure S6). Although the inconsistency test of the design-by-treatment interaction model was not necessary because the network meta-analysis of fatigue only consisted of 2 three-arm trials, very low heterogeneity was observed in pairwise comparisons (I^2^ = 0%; Figure S7). Egger’s test also showed no significance in the pooled estimate of fatigue (*p =* 0.248; Figure S8).

### 3.4. Memory and Concentration Impairments

Memory and concentration impairments are mainly discussed in terms of neurological sequelae. A four-node network meta-analysis of memory impairment was applied and included using three RCTs (*n* = 1166), two of which used the three-arm design and the other one used a two-arm design. The trials involved the following four treatments: NBO (*n* = 332), one-session HBO (*n* = 512), two-session HBO (*n* = 246), and three-session HBO (*n* = 76) treatments. No significant difference was observed in memory impairment among one-session (RR = 0.74), two-session (RR = 1.32), and three-session HBO (RR = 0.57) treatments compared with reference (Figure 3D). Two-session HBO treatment demonstrated a notably higher rate of memory impairment than one-session HBO treatment (RR=1.80, 95% CI: 1.01–3.19), and three-session HBO treatment did not lead to significantly decreased memory impairment compared with one-session (RR = 0.77) and two-session HBO (RR = 0.43) treatments. Although most findings were nonsignificant, some estimates (RR = 0.43 and RR = 0.57) raised concerns. Hence, we further performed SUCRA to obtain a possible priority. Two-session HBO treatment was still the worst treatment among the four interventions (Figure S9) The inconsistency test performed using the design-by-treatment interaction model showed no statistical significance in the network meta-analysis of memory impairment (*p =* 0.141; Figure S10). In addition, the Egger’s test also showed no significant finding in the consistency model of memory impairment (*p =* 0.296; Figure S11).

Four of the included trials (*n* = 1231) reported concentration impairments for NBO (*n* = 364), one-session HBO (*n* = 545), two-session HBO (*n* = 246), and three-session HBO (*n* = 76). No statistically significant differences were observed in concentration impairment among one-session (RR = 0.77), two-session (RR = 1.43), and three-session HBO (RR = 0.77) treatments compared with NBO (Figure 3E). Notably, two-session HBO treatment led to a higher rate of concentration impairment than the one-session HBO (RR = 1.85, 95% CI: 1.19 to 2.89). Although the three- and one-session HBOs (RR = 1.01) showed similar concentration impairment rates, the three-session HBO did not yield significantly lower rates of concentration impairment than two-session HBO treatment (RR = 0.54). SUCRA analysis again indicated that two-session HBO treatment was the worst treatment among the four interventions (Figure S12). The inconsistency test performed using the design-by-treatment interaction model showed no statistical significance in the network meta-analysis of concentration impairment (*p =* 0.866; Figure S13), and the Egger’s test also showed no significance in the consistency model of concentration impairment (*p =* 0.611), respectively (Figure S14).

## 4. Discussion

We included eight RCTs (*n* = 1785) in the present study, and the study revealed that, based on available evidence, HBO treatment might be a low-value treatment for CO poisoning. HBO treatments do not decrease mortality, memory impairment, and concentration impairment rates compared with NBO treatment. HBO treatments also failed to improve the headache rate. Moreover, two-session HBO treatment may cause more general fatigue than NBO treatment, and it led to a higher fatigue rate, memory impairment rate, and concentration impairment rate than one-session HBO treatment.

The present evidence is not completely consistent with previous observations [5,28], while our findings are in accordance with other recent studies [29,30]. Specifically, the recent studies indicate that HBO cannot effectively prevent delayed neurological sequelae after CO poisoning [29,30]. Moreover, more sessions of HBO do not result in a lower incidence of delayed neuropsychiatric sequelae after propensity score-matching analysis [30]. On the other hand, one of the previous observations supports the use of HBO in managing CO poisoning, but the favourable trend toward HBO is based on thiol/disulfide homeostasis rather than clinical outcomes [5]. The other previous observation indicated that HBO decreases the mortality rate, especially among patients younger than 20 years and having acute respiratory failure [28], whereas our analysis showed no statistically significant differences in mortality between HBO and NBO treatments. Based on all the available evidence on this topic, our synthesis does not agree with the findings of the previous observation. Although the findings of the previous observation may be based on possible mechanisms involving the metabolic rate and hypoxia, HBO might reduce mortality in patients with CO poisoning, because the brain and heart have high metabolic rates; thus, these organs are susceptible to hypoxia. CO poisoning first causes ischemic changes and then mortality. Moreover, CO poisoning induces immunologic and inflammatory damage to organs through the production of reactive oxygen species [7,28,31]. However, the effectiveness of HBO for mortality prevention in patients with CO poisoning might be limited by other stronger factors. A myocardial morphometric study by Fineschi, V. et al. might provide a potential reason for no significant difference in mortality between HBO and NBO [32]. On the basis of analysis of human cases and rates exposed to CO, the study found a characteristic of reperfusion injury that reoxygenation determines a necrosis typical of catecholamine myotoxicity. Myocardial cells injury may be most likely due to reoxygenation-related adrenergic stress rather than a direct CO poisoning or related anoxia. This may explain why NBO and HBO treatment had no statistically significant differences in mortality [32].

The reason why several scientific societies used HBO treatment to treat CO poisoning may be established by these studies. Kavakli, H.S. et al. described the effects on blood total oxidant–antioxidant levels in CO poisoning by analysis of 88 acute CO poisoning patients and 35 healthy adults as a control group. They took carboxyhemoglobin (COHb) levels and oxidative stress index levels in their decision of HBO treatment [33]. Cha, Y.S. et al. used four serum biomarkers expressed indicators of mitochondrial stress and oxidative stress. These four serum biomarkers were also used to place the patients in either favorable or poor outcome groups and reflect neuronal toxicity. They found that all four biomarkers decreased at 24 h post HBO therapy. In the poor-outcome group, the study described a significantly larger degree of change in these biomarkers after 24 h of HBO treatment. This result reflected an initial greater CO-associated stress, and the proportional response to HBO treatment [34]. Another study demonstrated oxidative stress and antioxidant parameter levels in patients with CO poisoning by analysis of serum and urine during the admission and after NBO and HBO treatment. CO poisoning increased lipid peroxidation immediately after the poisoning in this study. However, there is no significant effect on either NBO or HBO treatment. Only one HBO session may be safe in CO poisoning patients [35]. Although HBO alone appears to be not superior to NBO, initiation time point and the combination therapy of HBO may be worth further investigation. Actually, these two issues are mentioned in recent studies [36,37], but the abovementioned explanations are based on some studies without consideration of treatment timing or care bundle.

Other explanations and potential effect modifiers of our findings are as follows. Duration of CO exposure, CO level, and CO poisoning severity (coma at baseline) may influence the efficacy of HBO therapies. For instance, in the pooled analysis of mortality, we observed that the duration of CO exposure might influence the effects of HBO on mortality according to variation in CO exposure time across the three RCTs [11,12,27]. Patients in the study by Scheinkestel et al. (1999) [11] seemed to have a shorter CO exposure time (2.5 h) than those in the studies by Annane et al. (2011) [12] and Raphael et al. (1989) [27]. In the study by Scheinkestel et al. (1999) [11], the mortality rates were 2.88% and 3.45% in the HBO (3/104) and NBO groups (3/87), respectively. Conversely, we observed that NBO was associated with a lower mortality rate than HBO did in the studies by Annane et al. (2011) [12] and Raphael et al. (1989) [27], although the comparisons did not reach statistical significance. Taken together, the results indicate that patients with shorter CO exposure times may benefit from HBO therapy. In contrast to CO exposure time, no clear trends of the CO level or CO poisoning severity (coma at baseline) affecting mortality risks were observed between HBO and NBO treatments.

In addition to mortality, headache recovery is another important outcome in clinical practice, because it is the most common complaint of patients with CO poisoning [38]. The mechanism of headache with CO exposure is not exactly understood, and the probable reasons include tissue hypoxia, inflammation, vasodilatation, reactive oxygen species production, brain lipid peroxidation, and necrosis [28]. Past animal studies also showed that HBO therapy benefits brain lipid peroxidation [39,40]. Current evidence also does not support this presumption, because relevant studies and our synthesis did not observe significant improvement in headaches after patients received HBO treatment. In particular, the consistency model showed that the recovery rate of headaches in HBO treatment was not significantly lower than that in NBO treatment. Most studies in the consistency model showed a favorable trend toward NBO treatment in headache recovery, and only the trial by Annane et al. (2001) showed a favorable trend toward HBO treatment [13]. We believe that this phenomenon might be because of variation in the initial headache proportion across the RCTs. The average proportion of headache at baseline ranged from 62% to 89% [12,26,27], except in the trial by Annane et al. (2001) [13]. The initial headache proportions in the trial by Annane et al. were 41% and 48% in NBO and two-session HBO groups, respectively. Notably, two-session HBO treatment led to a lower headache recovery rate than one-session HBO treatment, with statistical significance. This result might be because of more severity and proportion of the initial headache in the two-session HBO group than in the one-session HBO group. The trial by Annane et al. (2011) [12] included more coma patients in the two-session HBO group than in the one-session HBO group; the trial by Raphael et al. (1989) [27] involved more patients with headache in the two-session HBO group than in the one-session HBO group at baseline.

Moreover, our study results do not support obvious benefits of HBO therapy for memory impairment and difficulty concentrating as compared with NBO therapy. Notably, two-session HBO therapy results in more memory impairment and difficulty concentrating than one-session HBO therapy. Baseline CO level, CO exposure time, and CO poisoning severity play roles in the efficacy of HBO therapies for this population, as mentioned earlier. The trials by Annane et al. (2011) [12] and Raphael et al. (1989) [27] involved patients with more severity in the two-session HBO group than in the one-session HBO group. Baseline severity should be an important factor leading to nonsignificant improvement in memory impairment and difficulty concentrating after HBO therapy. Two-session HBO therapy may not benefit coma patients with CO poisoning.

Hyperoxia might be another reasoning for how HBO therapies do not behave superior to NBO, although most of the included trials did not report hyperoxia. It is known that hyperoxia also leads to central nervous system symptoms after HBO therapy [41], and central nervous system oxygen toxicity is commonly caused by HBO therapy [42]. If HBO is inappropriately provided to patients, it will be harmful [42]. Hyperoxia should be taken into consideration when non-superiority of HBO therapies over NBO is observed in clinical practice.

Although we overcame some methodological limitations in the previous pairwise meta-analysis using contrast-based network meta-analysis, our study has certain limitations in pooled results. First, evidence quality might be concerns due to relatively high risk of bias in two of the included studies [2,13], while simply removing studies from meta-analysis due to quality would result in a form of selection bias [43]. To keep the completeness of evidence, the presence synthesis is based on all evidence on this topic. However, potential risk of bias ought to be taken into consideration before the application of these results. Second, our synthesis could not stratify the severity of CO poisoning and quality. Based on available data, patients with mild CO poisoning could not be distinguished from patients with severe CO poisoning. After noticing this limitation, we tried to consider baseline severity. Thus, we interpreted our results with this condition in the Discussion section. Third, beside sessions, HBO could be performed using various strategies. For instance, the trials included described various HBO chambers and durations, although we did not observe major inconsistency in the consistency models.

## 5. Conclusions

Collectively, our evidence indicates that HBO therapies might not be effective treatments for patients with CO poisoning, regardless of the type of outcomes in our synthesis. Therefore, clinicians should give more thought and consideration before recommending HBO to patients.

## Figures and Tables

**Table 1:** Characteristics of the included randomized controlled trials.

		Inclusion		Sex		CO Exposure	CO	Coma
Author	Location	Years	Group	(M/F)	Age	Time	Level	(*n*/N)
Annane	France	NR	NBO	ALL:	ALL:	ALL:	ALL:	ALL:
2001		(4 years)	1-HBO	149/158	49.7	5.9 h	22%	Unclear
Annane	France	1989 to	NBO	39/47	34	4 h	22%	3/86
2011		2000	1-HBO	80/114	35.1	4.4 h	22%	93/194
			2-HBO	44/61	37	3 h	26%	104/105
Ducasse	France	NR	NBO	8/5	31.6	<12 h	24%	9/13
1995			1-HBO	7/6	28.3	<12 h	23%	8/13
Hampson	USA	1995 to	1-HBO	10/8	47.2	2 h	22%	Unclear
2006		2002	2-HBO	4/8	43.1	2 h	24%	
Raphael	France	NR	NBO	91/79	35.6	6.2 h	22%	0/170
1989			1-HBO	143/175	36.4	7.1 h	23%	43/318
			2-HBO	56/85	37	5.3 h	25%	39/141
Scheinkestel	Australia	1993 to	NBO	67/20	34.8	2.5 h	22%	49/87
1999		1995	1-HBO	89/15	37.8	2.6 h	21%	53/104
Thom	USA	1989 to	NBO	18/14	39	NR	20%	Unclear
1995		1993	1-HBO	16/17	35		25%	
Weaver	USA	1992 to	NBO	54/22	36	NR	25%	38/76
2002		1999	3-HBO	54/22	35		25%	37/76

1-HBO, one-session hyperbaric oxygen therapy; 2-HBO, two-session hyperbaric oxygen therapy; 3-HBO, three-session hyperbaric oxygen therapy; CO, carbon monoxide; h, hours; M/F, male/female; NBO, normobaric oxygen therapy; NR, no report.

## Data Availability

All data generated or analyzed during this study are included in this published article.

## Supplementary Materials

The following supporting information can be downloaded at: https://www.mdpi.com/article/10.3390/healthcare10071311/s1. Supplementary Materials S1. Search strategy; Table S1. Risk of bias; Figure S1. Inconsistency test of mortality; Figure S2. Small study effect test for mortality; Figure S3. Probability ranking of headache recovery; Figure S4. Inconsistency test of headache recovery; Figure S5. Small study effect test for headache recovery; Figure S6. Probability ranking of fatigue; Figure S7. Heterogeneity test of fatigue; Figure S8. Small study effect test for fatigue; Figure S9. Surface under the cumulative ranking curve of memory impairment; Figure S10. Inconsistency test of memory impairment; Figure S11. Small study effect test for memory impairment; Figure S12. Surface under the cumulative ranking curve of concentration impairment; Figure S13. Inconsistency test of concentration impairment; Figure S14. Small study effect test for concentration impairment.

## Abbreviations

CI	confidence intervals
CO	carbon monoxide
HBO	hyperbaric oxygen
NBO	normobaric oxygen
RCT	randomized clinical trial
RR	risk ratio
SUCRA	surface under the cumulative ranking
