# Supplementary material for: Should We Use Hyperbaric Oxygen for Carbon Monoxide Poisoning Management? A Network Meta-Analysis of Randomized Controlled Trials
Source: Healthcare (Basel). 2022 Jul 14;10(7):1311. doi: 10.3390/healthcare10071311 (PMC9318730; doi:10.3390/healthcare10071311)
Supplement: Supplementary file 1 [file healthcare-10-01311-s001.zip › healthcare-1667517-supplementary.pdf]

Healthcare

Should we use hyperbaric oxygen for treating carbon monoxide poisoning management?

A systematic review with network meta-analysis

(Supplementary Files)

Authors:

Yu-Wan Ho, M.D. [1], Ping-Yen Chung, M.D. [1], Sen-Kuang Hou, M.D., Ph.D., Director[1], Ming-Long Chang, M.D. [1], Yi-No Kang, Consultant [1,2,3,4,5,]

Affiliations:

1. Department of Emergency Medicine, Taipei Medical University Hospital, Taipei, Taiwan
2. Evidence-Based Medicine Center, Wan Fang Hospital, Taipei Medical University, Taipei, Taiwan
3. Research Center of Big Data and Meta-Analysis, Wan Fang Hospital, Taipei Medical University, Taipei, Taiwan
4. Cochrane Taiwan, Taipei Medical University, Taipei, Taiwan
5. Institute of Health Policy and Management, College of Public Health, National Taiwan University, Taipei, Taiwan

Supplementary File

Material S1. Search strategy

Table S1. Risk of bias

Figure S1. Inconsistency test of mortality

Figure S2. Small study effect test for mortality

Figure S3. Probability ranking of headache recovery

Figure S4. Inconsistency test of headache recovery

Figure S5. Small study effect test for headache recovery

Figure S6. Probability ranking of fatigue

Figure S7. Heterogeneity test of fatigue

Figure S8. Small study effect test for fatigue

Figure S9. Surface under the cumulative ranking curve of memory impairment

Figure S10. Inconsistency test of memory impairment

Figure S11. Small study effect test for memory impairment

Figure S12. Surface under the cumulative ranking curve of concentration impairment

Figure S13. Inconsistency test of concentration impairment

Figure S14. Small study effect test for concentration impairment

Material S1
Search strategy

Primary search steps:

#1. Carbon monoxide

#2. Carbon monoxide intoxication

#3. co poisoning

#4. Carbon monoxide poisoning

#5. #1 OR #2 OR #3 OR #4

#6. Hyperbaric oxygenation therapy

#7. Hyperbaric oxygenation

#8. Hyperbaric oxygen

#9. Hyperbaric O2

#10. High pressure oxygen

#11. #6 OR #7 OR #8 OR #9 OR #10

#12. #5 AND #11

Final syntax in Embase:

(('carbon monoxide intoxication'/exp OR 'carbon monoxide intoxication' OR 'carbon monoxide poisoning' OR 'co intoxication' OR 'coal gas poisoning' OR 'cointoxication' OR 'intoxication, carbon monoxide' OR 'poisoning, carbon monoxide' OR 'co poisoning') AND ('hyperbaric oxygen therapy'/exp OR 'high pressure oxygen' OR 'high tension o2' OR 'high tension oxygen' OR 'hyperbaric medicine' OR 'hyperbaric o2' OR 'hyperbaric oxygen therapy' OR 'hyperbaric oxygenation' OR 'oxygen, hyperbaric' OR 'hyperbaric oxygen')) AND ('controlled clinical trial'/de OR 'double blind procedure'/de OR 'prospective study'/de OR 'randomized controlled trial'/de OR 'randomized controlled trial (topic)'/de)

Final syntax in New PubMed:

(Carbon monoxide OR Carbon monoxide intoxication OR co poisoning OR Carbon monoxide poisoning) AND (Hyperbaric oxygenation therapy OR Hyperbaric oxygenation OR Hyperbaric oxygen OR Hyperbaric O2 OR High pressure oxygen) Filters: Clinical Trial, Randomized Controlled Trial Sort by: Most Recent

Final syntax in Web of Science:

TS=(Carbon monoxide OR Carbon monoxide intoxication OR co poisoning OR Carbon monoxide poisoning) AND TS=(Hyperbaric oxygenation therapy OR Hyperbaric oxygenation OR Hyperbaric oxygen OR Hyperbaric O2 OR High pressure oxygen) AND TS=(random*)

Timespan: All years. Databases: WOS, BIOSIS, MEDLINE, RSCI.

Search language=Auto

Table S1

Risk of bias summary

Study	1	2	3	4	5	6	7
Annane (2001)	Some concerns	High risk	High risk	High risk	Low risk	High risk	Some concerns
Annane (2011)	Low risk	Low risk	High risk	Low risk	Low risk	Low risk	Low risk
Ducasse (1995)	Unclear	Unclear	High risk	Low risk	High risk	Low risk	Low risk
Hampson (2006)	Low risk	Low risk	Some concerns	High risk	Low risk	Some concerns	Low risk
Raphael (1989)	Low risk	Low risk	Some concerns	High risk	Low risk	Low risk	High risk
Scheinkestel (1999)	Some concerns	Some concerns	Some concerns	Low risk	Low risk	Low risk	Low risk
Thom (1995)	Some concerns	Some concerns	High risk	High risk	High risk	Some concerns	Low risk
Weaver (2002)	Low risk	Low risk	Low risk	Low risk	Low risk	Low risk	Low risk

1 sequence generation; 2 allocation concealment; 3 blinding of participants and personnel; 4 blinding of outcome assessment; 5 incomplete outcome data; 6 selective reporting; 7 other bias.

Figure S1

Inconsistency test of mortality

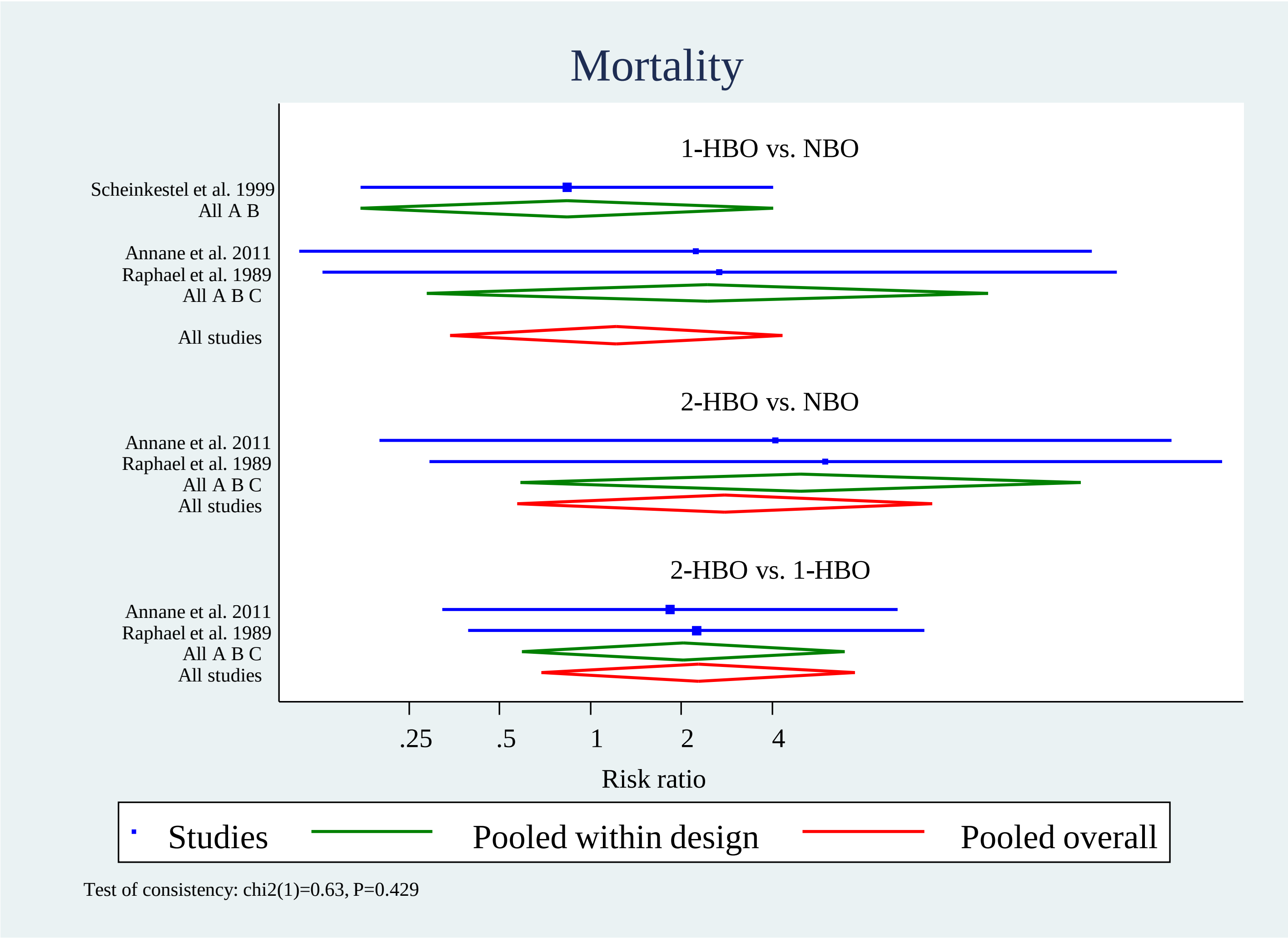

Figure S2

Small study effect test for mortality

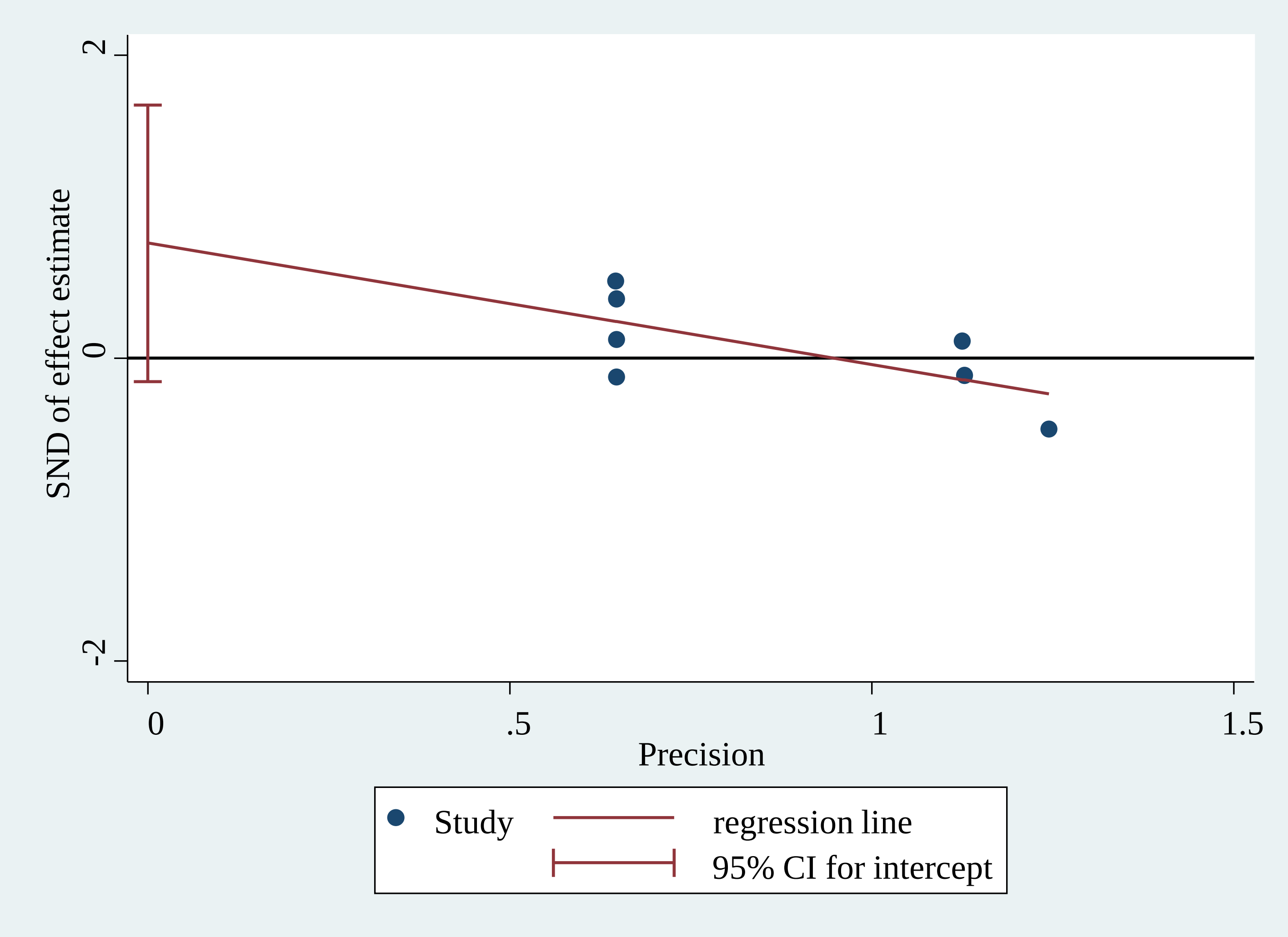

Egger's test for small-study effects:
Regress standard normal deviate of intervention
effect estimate against its standard error

Number of studies = 7 Root MSE = .2684

Std_Eff	Coef.	Std. Err.	t	P>|t|	[95% Conf. Interval]	
slope	-.8009956	.3913759	-2.05	0.096	-1.807059	.205068
bias	.7585251	.3551887	2.14	0.086	-.1545165	1.671567

Figure S3

Probability ranking of headache recovery

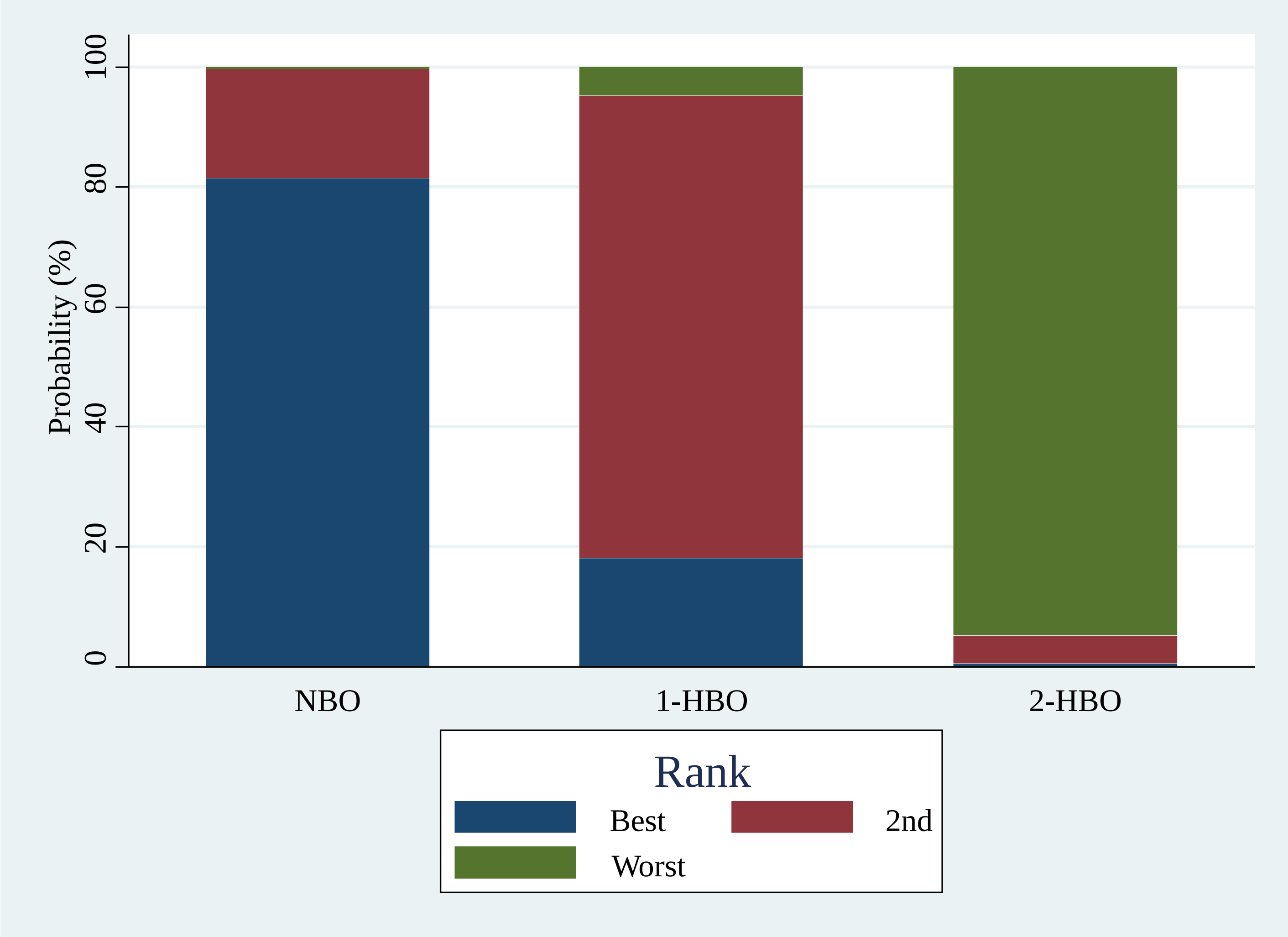

Figure S4

Inconsistency test of headache recovery

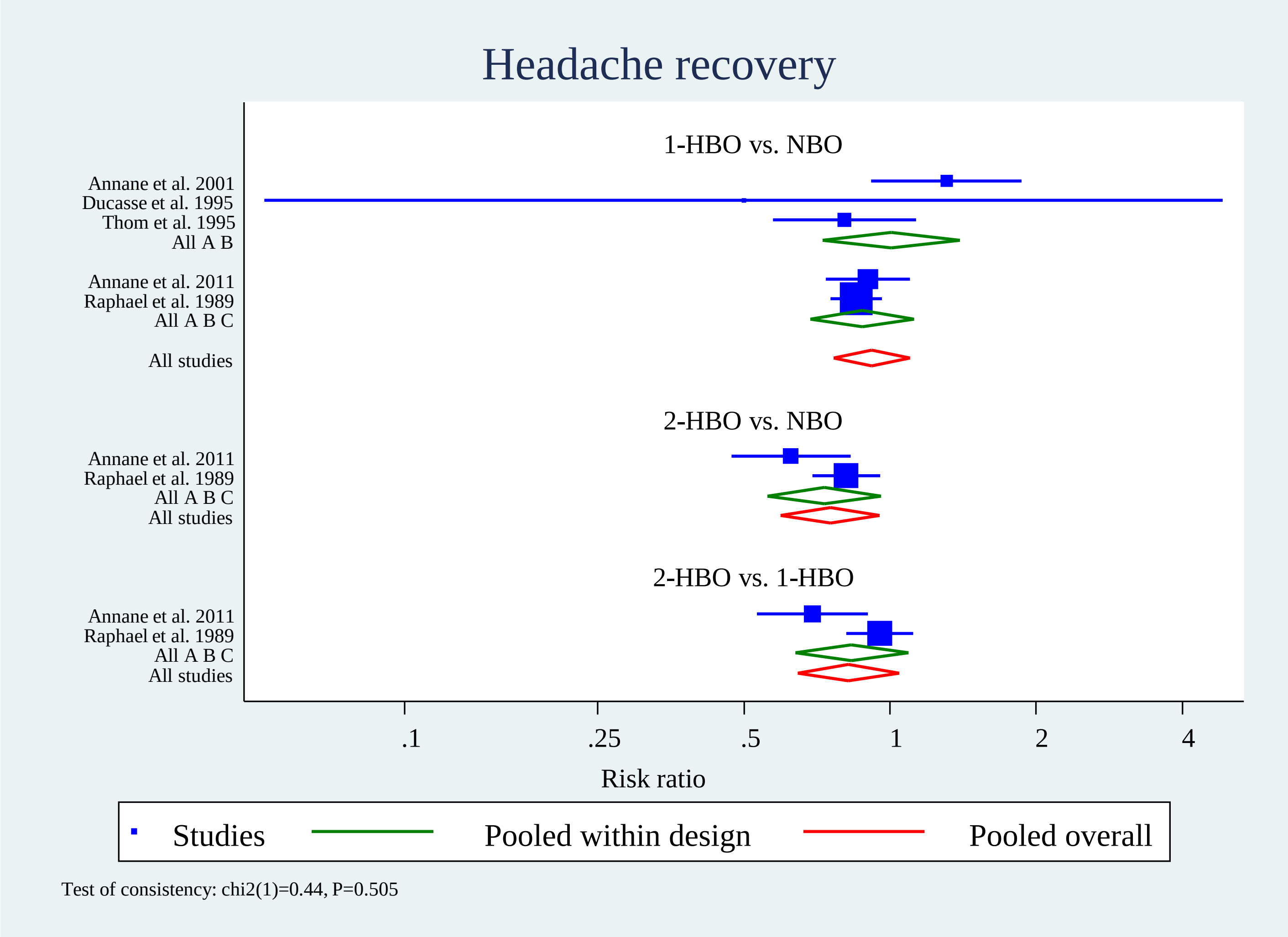

Figure S5

Small study effect test for headache recovery

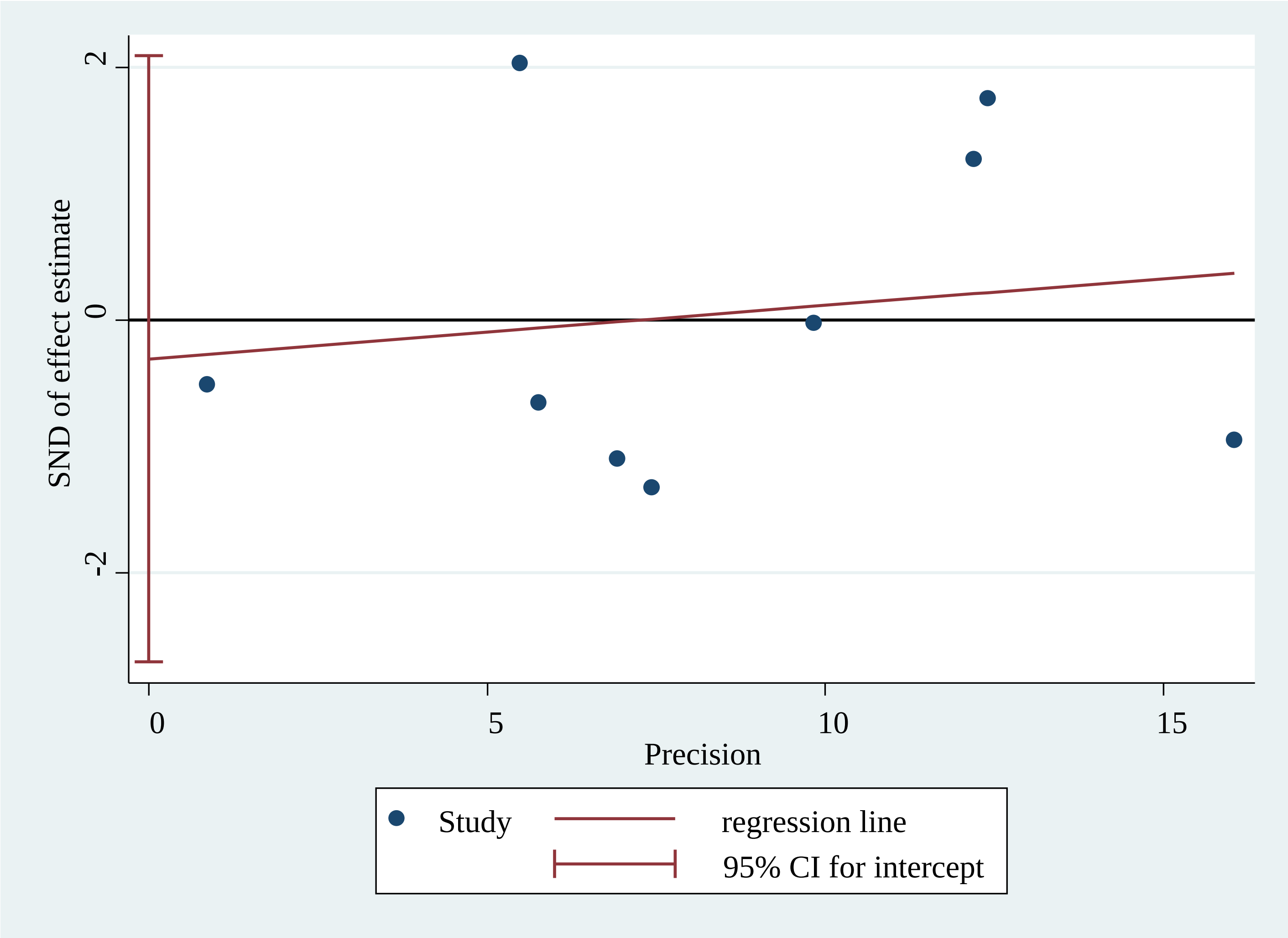

Egger's test for small-study effects:
Regress standard normal deviate of intervention
effect estimate against its standard error

Number of studies = 9   Root MSE = 1.366

Std_Eff	Coef.	Std. Err.	t	P>|t|	[95% Conf. Interval]	
slope	.0423027	.1059777	0.40	0.702	-.2082948	.2929002
bias	-.3055131	1.014111	-0.30	0.772	-2.703505	2.092479

Figure S6
Probability ranking of fatigue

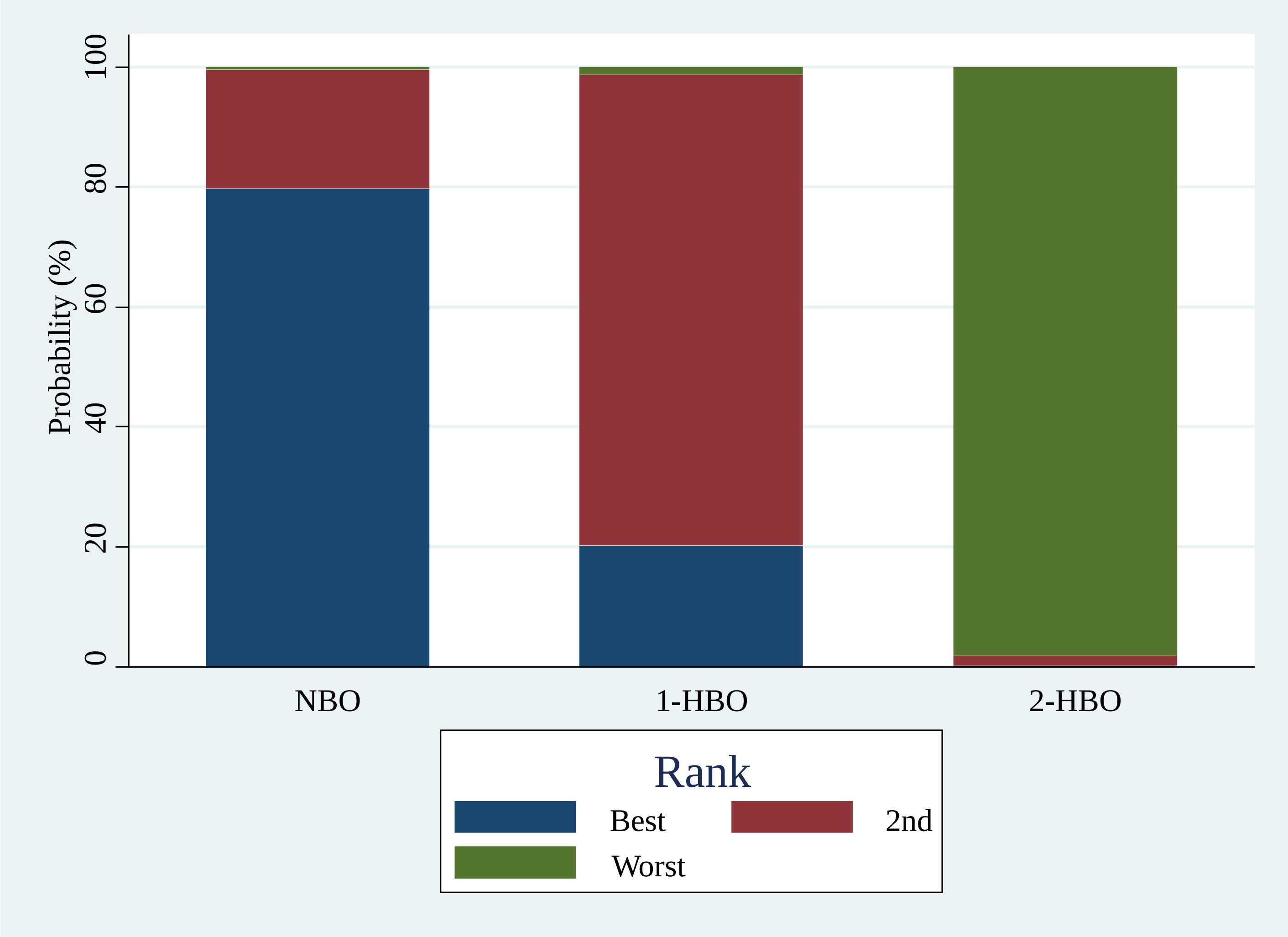

Figure S7

Heterogeneity test of fatigue

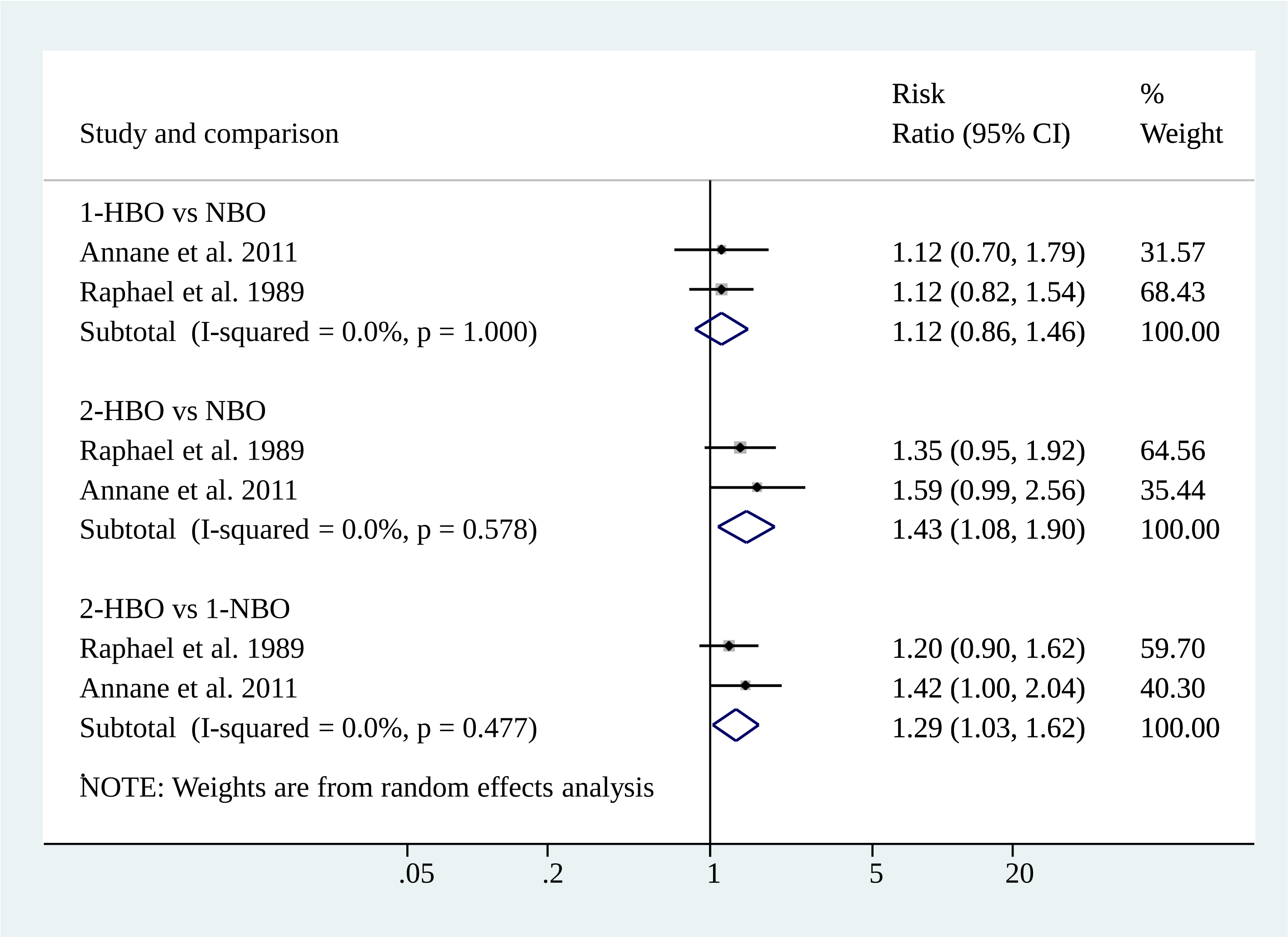

Figure S8

Small study effect test for fatigue

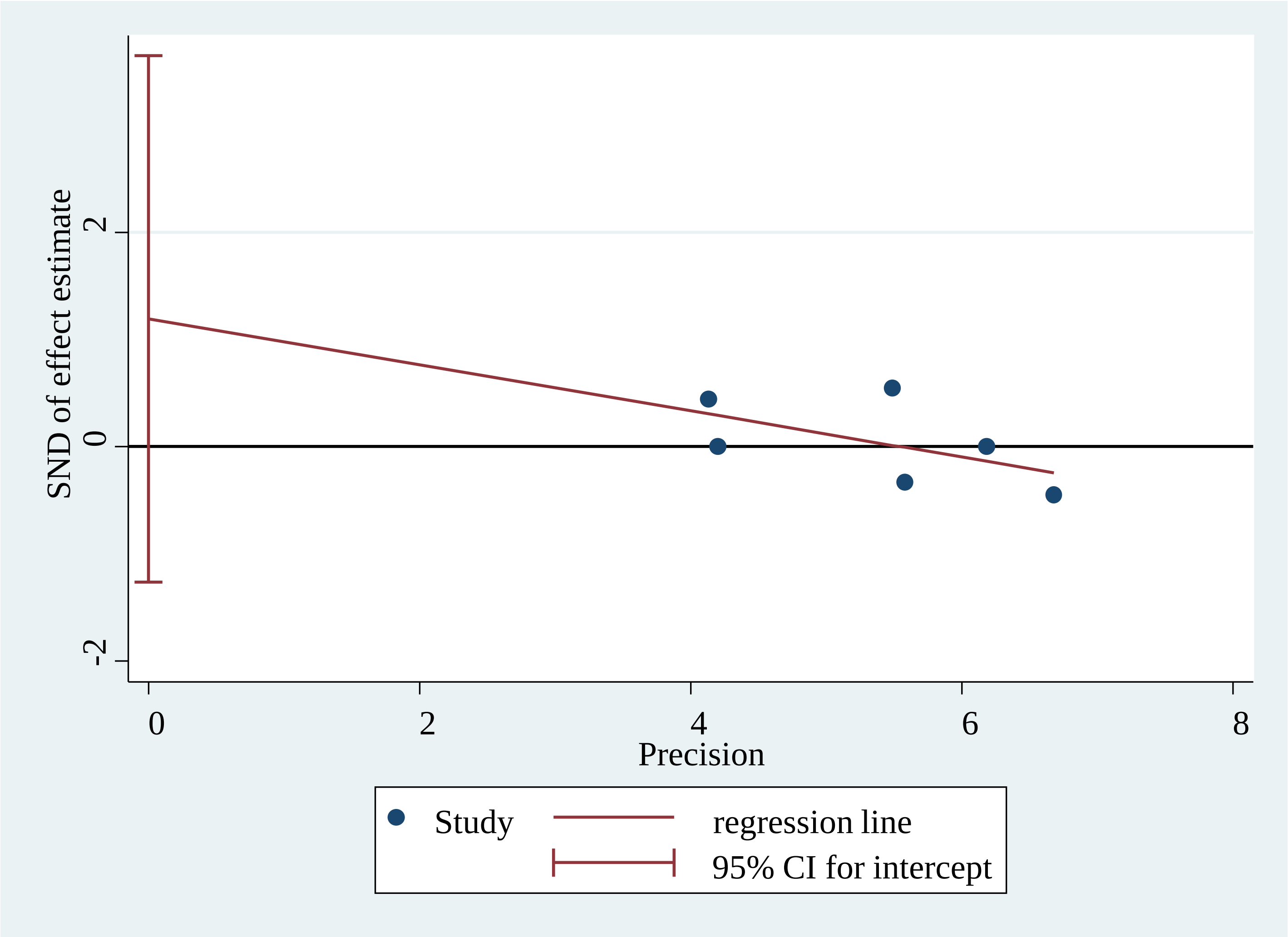

Egger's test for small-study effects:
Regress standard normal deviate of intervention
effect estimate against its standard error

Number of studies = 6 Root MSE = .3739

Std_Eff	Coef.	Std. Err.	t	P>|t|	[95% Conf. Interval]	
slope	-.2152102	.1618617	-1.33	0.254	-.6646103	.2341899
bias	1.193058	.883828	1.35	0.248	-1.260842	3.646958

Figure S9

Surface under the cumulative ranking curve of memory impairment

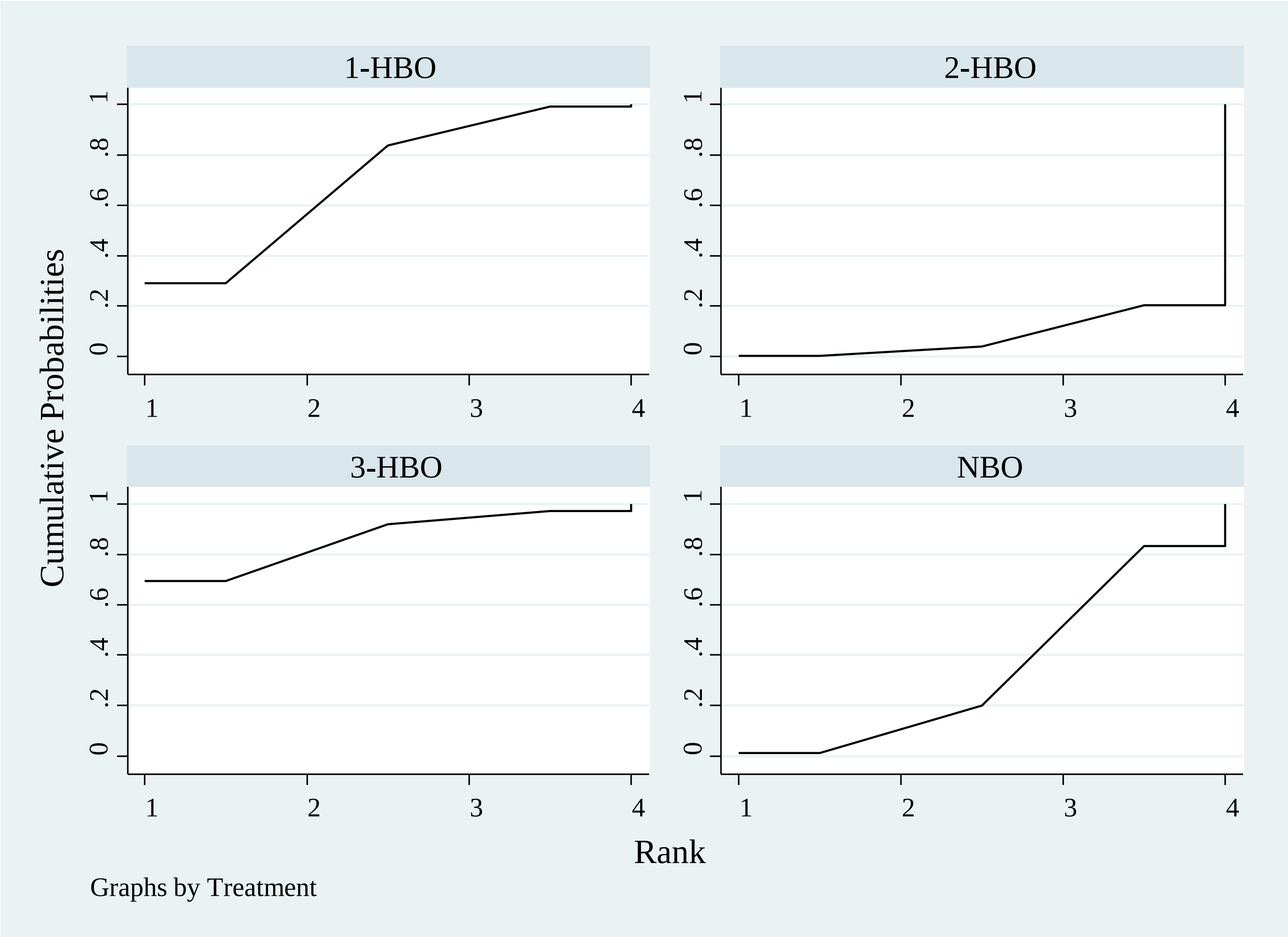

Treatm~t	SUCRA	PrBest	MeanRank
NBO	34.8	1.2	3.0
1-HBO	70.5	29.0	1.9
2-HBO	8.4	0.3	3.7
3-HBO	86.3	69.5	1.4

Figure S10

Inconsistency test of memory impairment

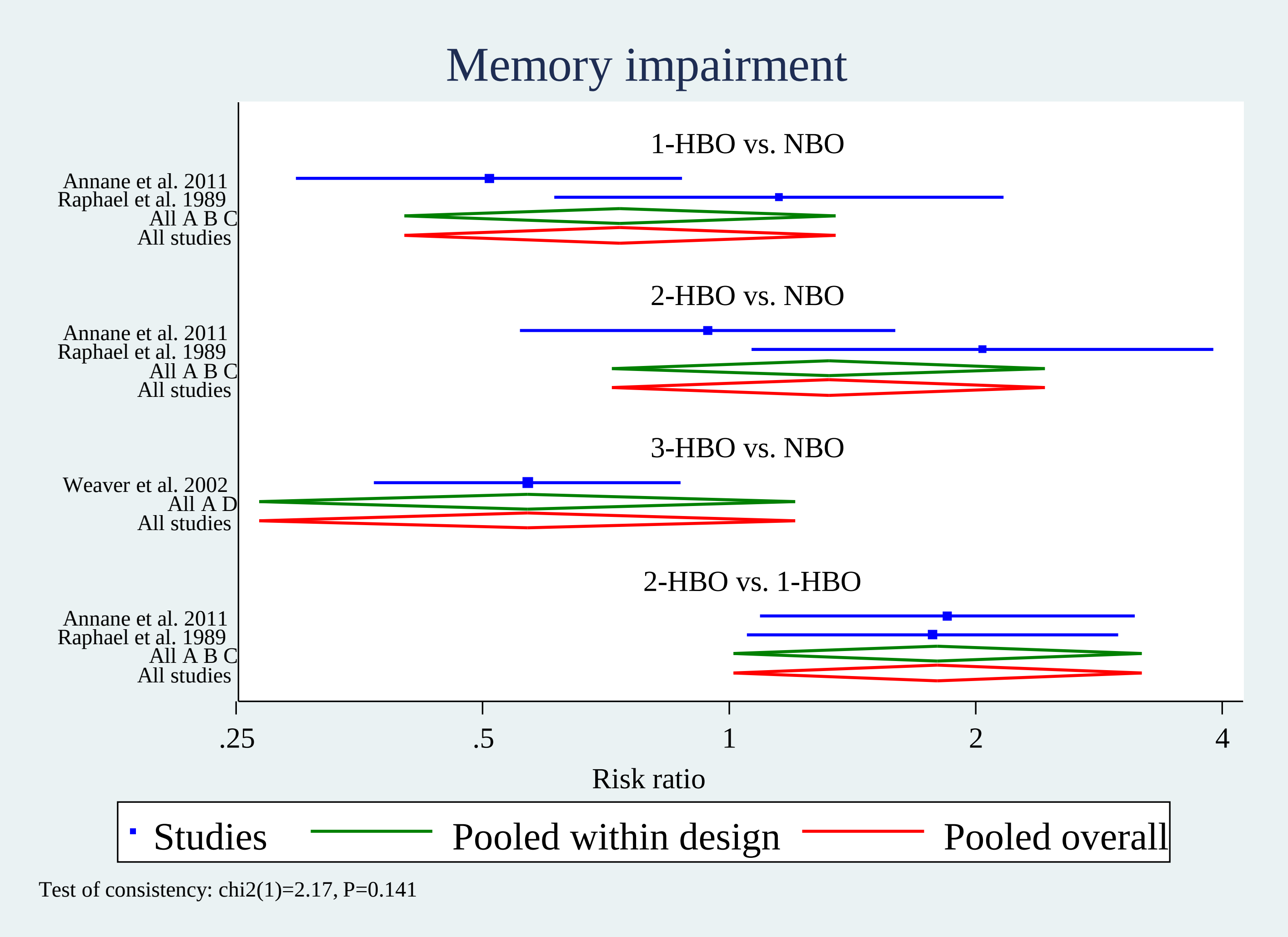

Figure S11

Small study effect test for memory impairment

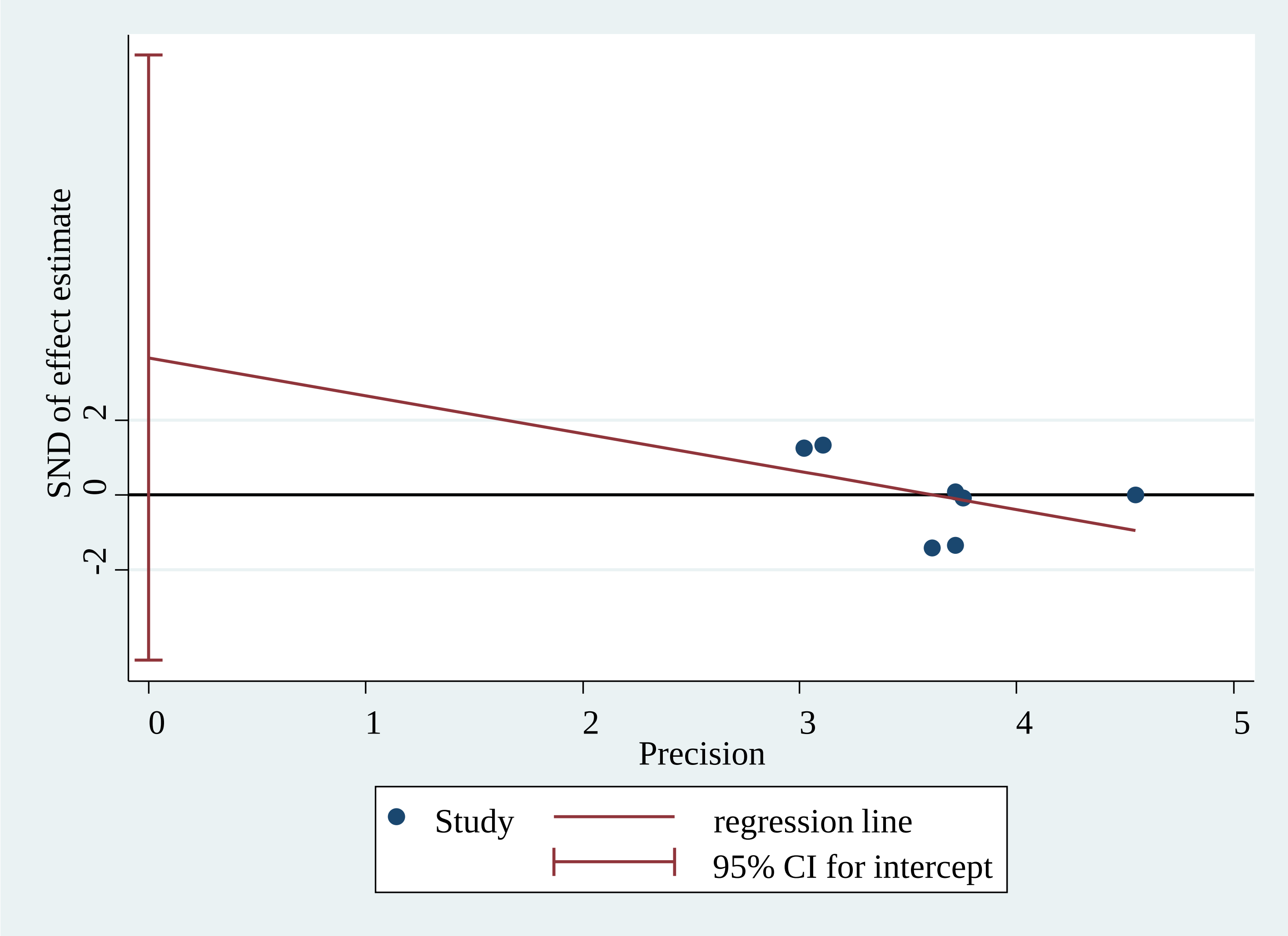

Egger's test for small-study effects:
Regress standard normal deviate of intervention
effect estimate against its standard error

Number of studies = 7 Root MSE = 1.052

Std_Eff	Coef.	Std. Err.	t	P>|t|	[95% Conf. Interval]	
slope	-1.01171	.8543914	-1.18	0.290	-3.207993	1.184573
bias	3.6543	3.135598	1.17	0.296	-4.406012	11.71461

Figure S12

Surface under the cumulative ranking curve of concentration impairment

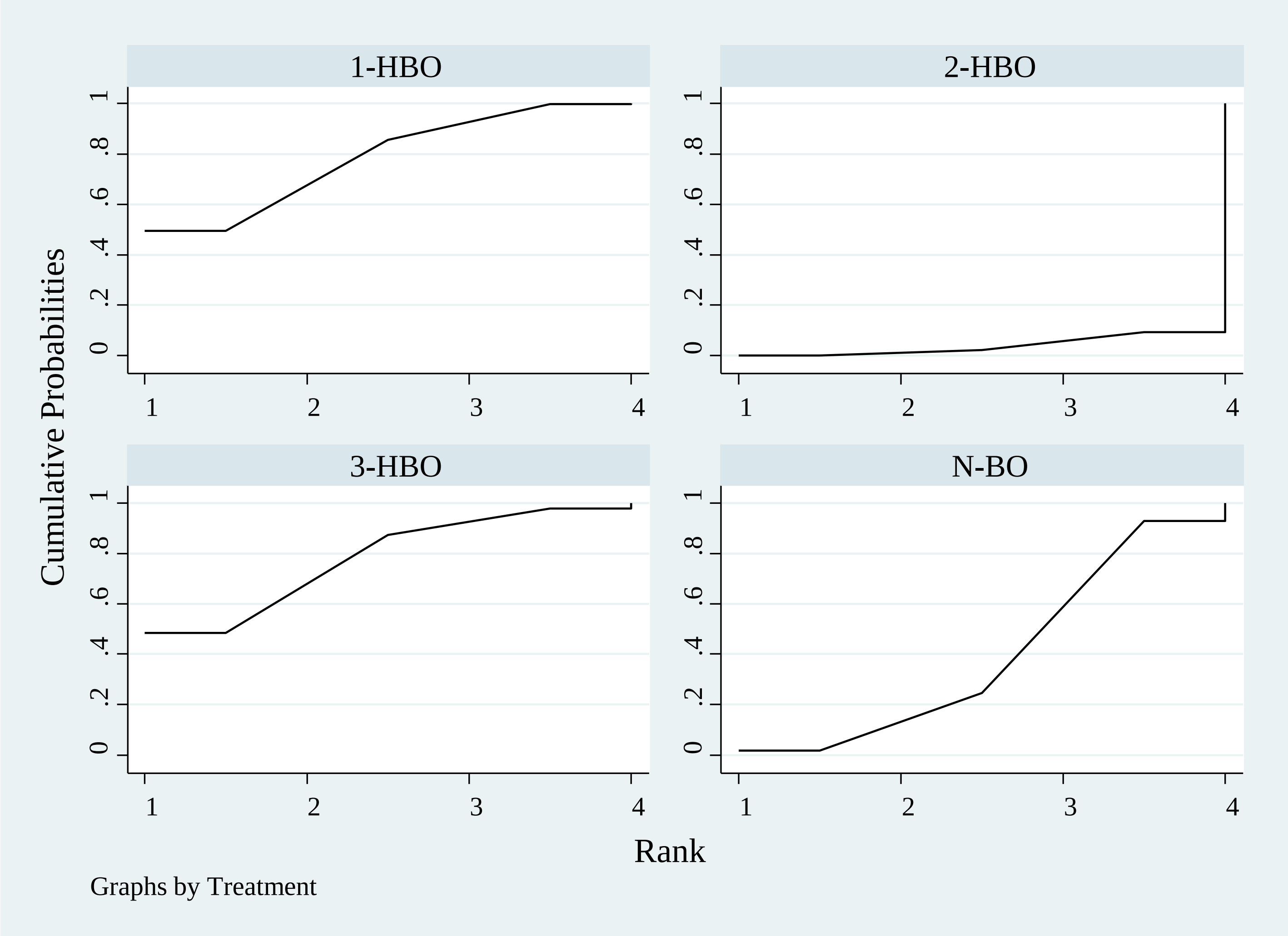

Treatm~t	SUCRA	PrBest	MeanRank
NBO	39.7	1.8	2.8
1HBO	78.3	49.4	1.7
2HBO	4.0	0.1	3.9
3HBO	78.0	48.6	1.7

Figure S13

Inconsistency test of concentration impairment

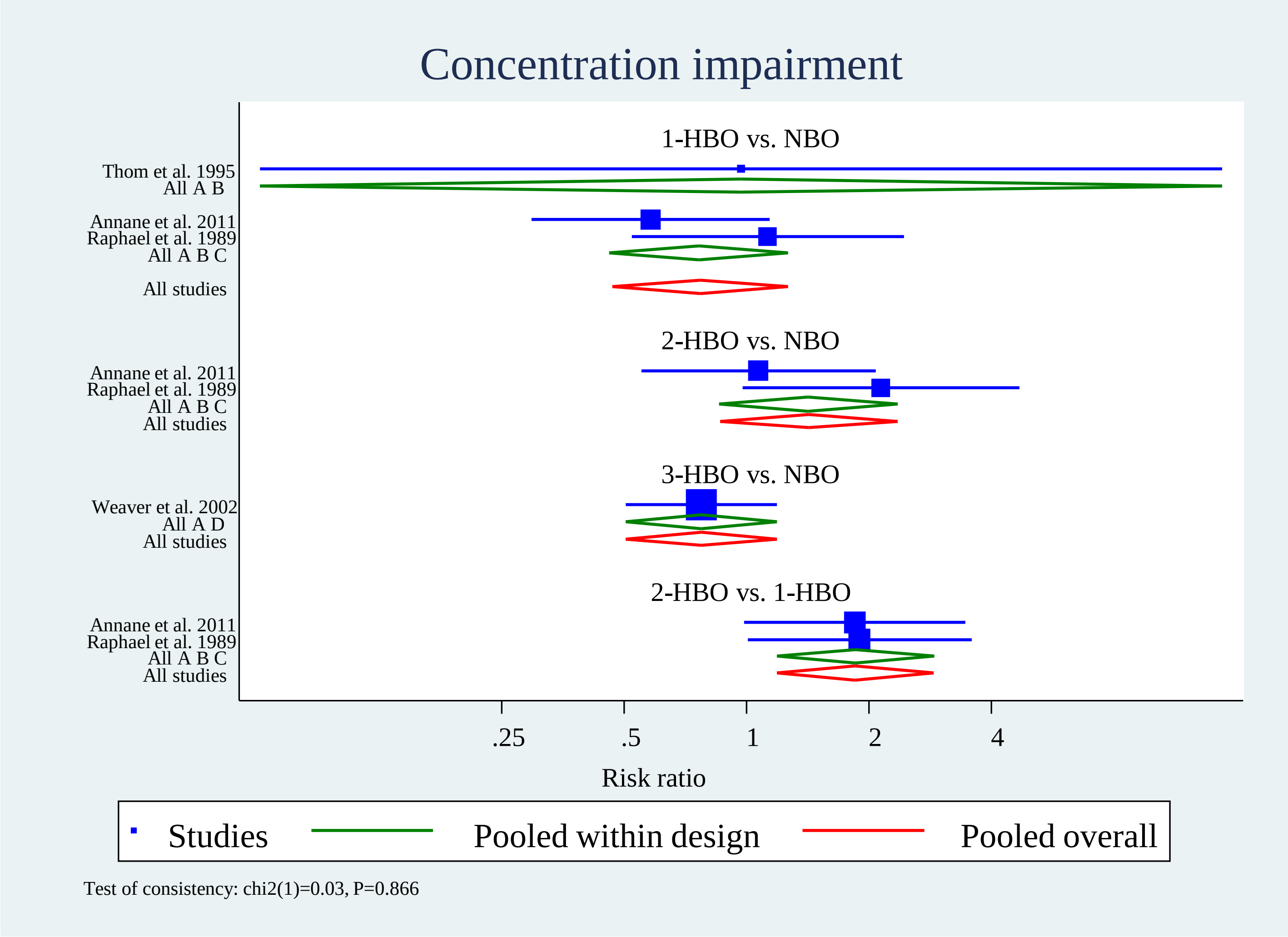

Figure S14
Small study effect test for concentration impairment

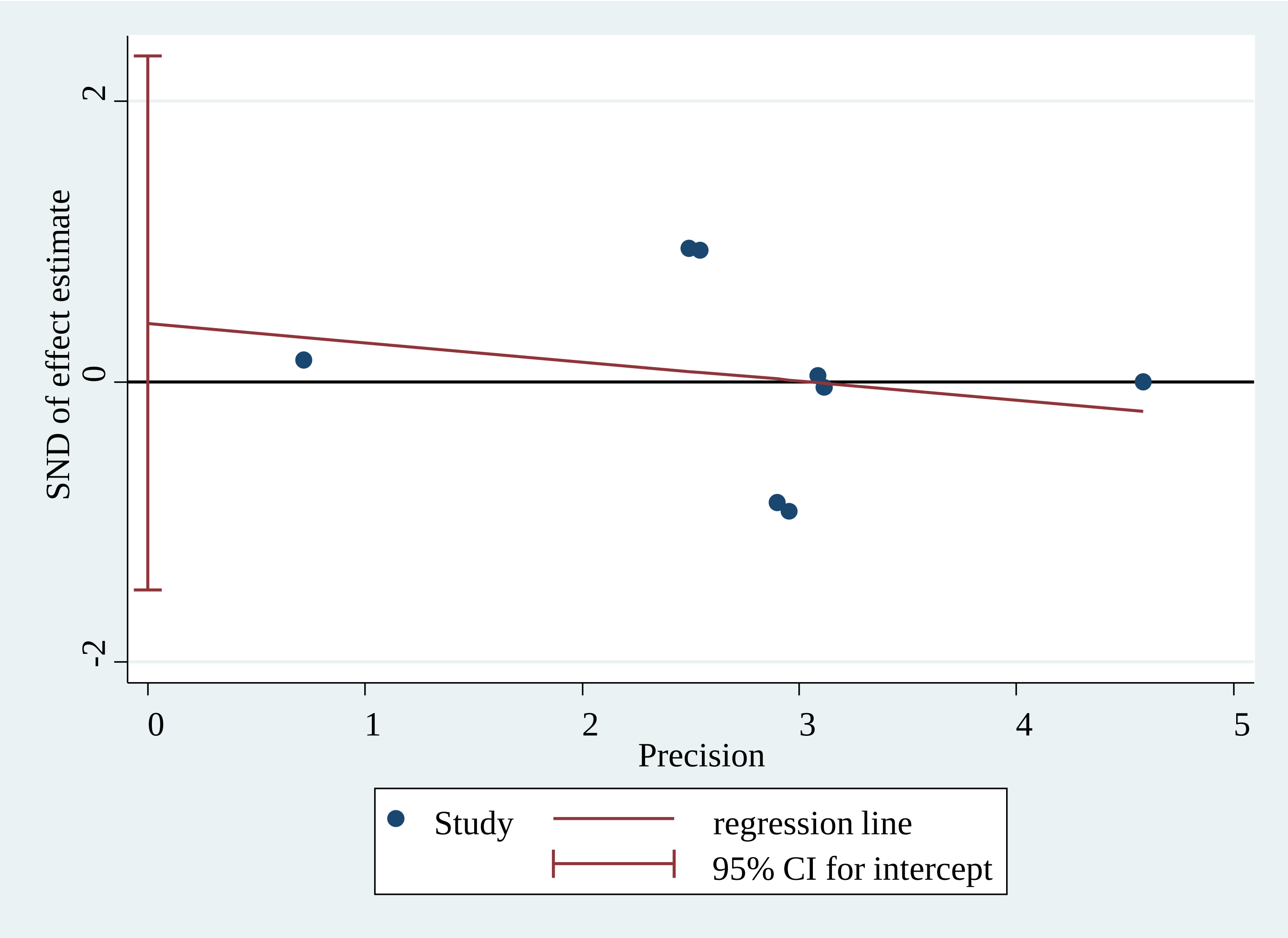

Egger's test for small-study effects:
Regress standard normal deviate of intervention
effect estimate against its standard error

Number of studies = 8 Root MSE = .7361

Std_Eff	Coef.	Std. Err.	t	P>|t|	[95% Conf. Interval]	
slope	-.1375619	.2620798	-0.52	0.618	-.7788481	.5037243
bias	.4169739	.7784829	0.54	0.611	-1.487905	2.321853
